# Single-session photon-counting CT protocol for simultaneous screening of coronary and carotid artery disease and lung pathology

**DOI:** 10.1093/ehjimp/qyag074

**Published:** 2026-04-18

**Authors:** Carmelo De Gori, Alberto Aimo, Luna Latorre, Denisa Simona Zai, Filippo Bruschi, Mariaelena Occhipinti, Matilda Muca, Francesca Pignatelli, Michele Emdin, Alberto Clemente

**Affiliations:** Radiology Department, Fondazione Toscana Gabriele Monasterio, Pisa e Massa, Italy; Cardiology Department, Fondazione Toscana Gabriele Monasterio, via G. Moruzzi 1, Pisa 56124, Italy; Interdisciplinary Center for Health Sciences, Scuola Superiore Sant’Anna, Piazza Martiri della Libertà, 33, Pisa 56127, Italy; University of Pisa, Italy; Radiology Department, Fondazione Toscana Gabriele Monasterio, Pisa e Massa, Italy; Radiology Department, Fondazione Toscana Gabriele Monasterio, Pisa e Massa, Italy; Radiology Department, Fondazione Toscana Gabriele Monasterio, Pisa e Massa, Italy; Radiology Department, Fondazione Toscana Gabriele Monasterio, Pisa e Massa, Italy; Radiology Department, Fondazione Toscana Gabriele Monasterio, Pisa e Massa, Italy; Cardiology Department, Fondazione Toscana Gabriele Monasterio, via G. Moruzzi 1, Pisa 56124, Italy; Interdisciplinary Center for Health Sciences, Scuola Superiore Sant’Anna, Piazza Martiri della Libertà, 33, Pisa 56127, Italy; Radiology Department, Fondazione Toscana Gabriele Monasterio, Pisa e Massa, Italy

**Keywords:** photon-counting CT, coronary CT angiography, carotid artery imaging, lung cancer screening, calcium scoring, cardiovascular prevention

## Abstract

**Aims:**

Preventive imaging for cardiothoracic risk is fragmented across separate pathways. Photon-counting CT (PCCT) may consolidate coronary, aortic–carotid, and pulmonary assessment into a single session while maintaining diagnostic performance and controlling dose.

**Methods and results:**

ACTA is an ongoing, general-practitioner–initiated, risk-enriched screening study (age 45–75 years; diabetes ≥10 years, current/recent smoking, or Framingham hard coronary artery disease risk ≥10%). The protocol integrates non-contrast calcium scoring, ultra-high-resolution coronary CT angiography, a high-pitch thoraco-cervical sweep, and brief late iodine enhancement (LIE). Incidental findings trigger predefined, guideline-concordant referrals. The primary endpoint is the prevalence of obstructive (≥50%) and/or extensive (≥2-vessel) coronary artery disease. This interim analysis includes participants imaged 11 January to 21 June 2025. Of 223 invited, 172 underwent PCCT; all completed without complications. Mean dose-length product was ∼740 mGy·cm; the effective dose was 12.6 mSv (IQR 10.5–17.1 mSv), and weight-adapted contrast equalled ∼0.44–0.48 gI/kg. Any coronary atherosclerosis was present in 129/172 (75%); the primary endpoint was met in 98/172 (57%). Carotid plaques occurred in 94/170 (55%); lung-RADS 3–4 in 16/172 (9%); emphysema in 41/172 (24%). Management actions included invasive coronary angiography in 8/172 (5%), targeted vascular and pulmonary referrals, initiation/intensification of prevention therapies, and structured follow-up. Incidental extracardiac findings were common but managed via protocolized pathways.

**Conclusion:**

Single-session PCCT feasibly consolidates comprehensive cardiothoracic assessment with controlled radiation/iodine exposure and structured downstream care. Preliminary yield suggests actionable information in high-risk, asymptomatic adults; ongoing follow-up and prespecified economic analyses will determine clinical outcomes and cost-effectiveness.

## Graphical Abstract

**Graphical Abstract:**
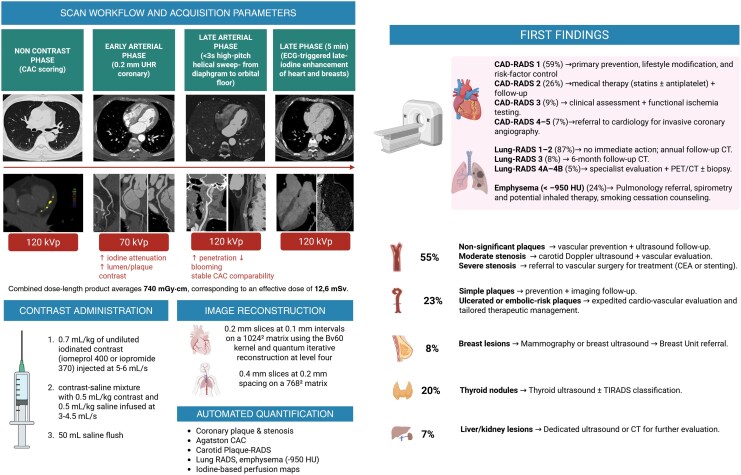

## Background

Cardiovascular and pulmonary diseases are leading causes of premature morbidity and mortality and share major, modifiable risks, particularly smoking.^1^ Important diagnostic gaps persist in asymptomatic, high-risk adults: subclinical and non-obstructive coronary atherosclerosis is often under-recognized yet prognostically meaningful^2,3^; carotid plaque burden that reclassifies risk is rarely assessed alongside coronary status^4,5^; and lung cancer screening and thoracic evaluation typically occur on separate pathways, increasing attrition and delaying preventive action.^6,7^ Photon-counting CT (PCCT) directly counts and energy-resolves incoming X-ray photons, enabling virtual mono-energetic images, material decomposition, and ultra-high-resolution (UHR) reconstructions with reduced electronic noise and blooming.^8^ These capabilities offer a practical route to address the technical and workflow limitations above within a single examination.

The ACTA study (‘Screening Coronary and Carotid Atherosclerosis and Chest Cancer by Computed Tomography Angiography with Photon-Counting Detector Technology’) is a risk-enriched, general practitioner (GP)-initiated study aligned with current prevention frameworks: eligibility maps to contemporary cardiovascular prevention guidance,^9^ and thoracic findings are managed within established lung cancer screening/surveillance pathways.^6^ The study tests a single-session PCCT workflow generating actionable information on the heart, lungs, aorta, and carotids, and is not meant as a replacement for validated screening programmes. Herein, we describe the PCCT protocol, report the preliminary findings about clinical yield, and provide technical details.

## Methods

### Study design

**Figure 1:**
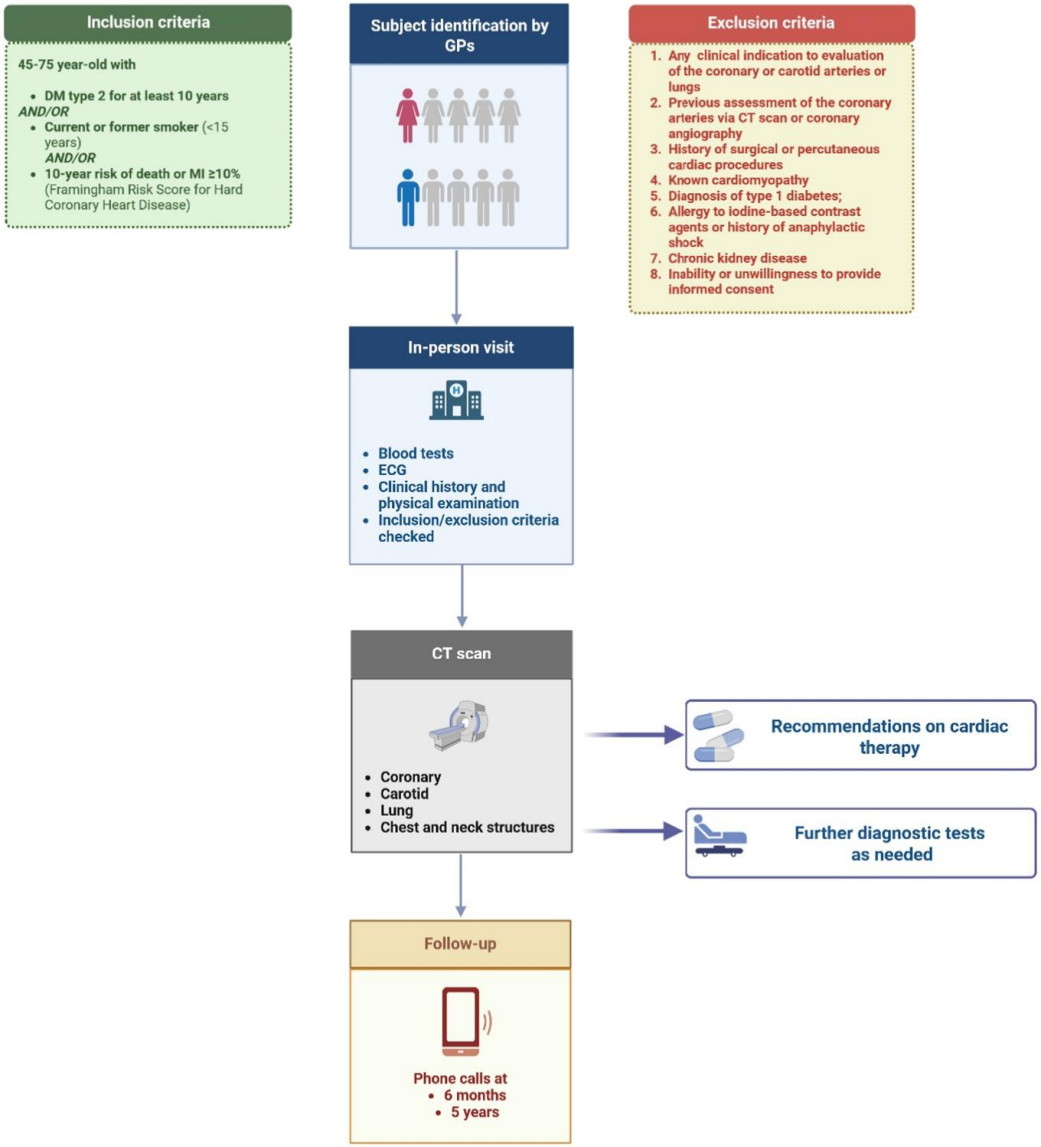
Overview of the ACTA screening protocol. See text for details. CT, computed tomography; ECG, electrocardiogram.

ACTA is an ongoing screening initiative targeting individuals at high risk for atherosclerotic disease and/or lung cancer using PCCT. The study design is schematized in *Figure 1*. GPs in the province of Pisa are instructed to invite individuals aged 45–75 years meeting at least one of the following: (i) type 2 diabetes mellitus for ≥10 years; (ii) current smoker or former smoker who quit <15 years prior; or (iii) no diabetes and no smoking history but a 10-year risk of death or myocardial infarction ≥10% by the Framingham Risk Score for Hard Coronary Heart Disease.^10^ Exclusion criteria are (i) signs, symptoms, or clinical indications warranting targeted evaluation of the coronary or carotid arteries or lungs; (ii) prior coronary evaluation; (iii) prior surgical or percutaneous cardiac procedures; (iv) cardiomyopathy; (v) iodine-contrast allergy or anaphylaxis; (vi) chronic kidney disease; (vii) inability or unwillingness to provide informed consent; and (viii) pregnancy. Subjects meeting all inclusion and no exclusion criteria undergo an in-person visit and the PCCT scan. A comprehensive report is then provided detailing results and recommendations for further tests, specialist consultations, treatment modification, and follow-up. Recommendations for cardiology management are based on the European Society of Cardiology prevention guidelines.^9^ Pulmonary nodules are managed according to Lung-RADS-based recommendations^9^; emphysema, interstitial changes, or bronchiectasis prompt formal pulmonary function testing and pneumology consultation when moderate-to-severe or symptomatic disease is present. Carotid disease with suspected ≥50% stenosis or ulcerated plaque prompts Doppler ultrasound and vascular consultation; aortic diameters meeting pre-specified thresholds prompt vascular referral and surveillance. Suspected breast lesions on the delayed cardiac phase are referred to specialist examination. Thyroid nodules are referred for dedicated ultrasound when ≥1 cm or when imaging suggests high-risk morphology. Adrenal nodules ≥1 cm are referred for adrenal-protocol CT or magnetic resonance imaging and endocrine assessment. Solid or enhancing renal masses are referred to urology, while hepatic lesions with non-cystic features are referred to hepatology. Findings judged definitively benign (e.g. simple cysts, benign bone islands, calcified granulomas) do not prompt further testing. All referrals and follow-up actions are communicated to the GP; the study team is available for clarification or re-evaluation as needed.

To address cost-effectiveness and potential over-medicalization, we will conduct a pre-specified economic evaluation comparing the single-session PCCT strategy with usual care (separate, indication-based pathways, and standard screening programmes) over a 5-year horizon.

Here we report a preliminary analysis from 11 January 2025 to 21 June 2025; enrolment will continue until 560 participants have undergone PCCT (anticipated completion November 2026). The primary endpoint is the prevalence of obstructive (≥50% stenosis) and/or extensive (≥2-vessel) CAD. Sample size was calculated for an expected 50% prevalence with 95% confidence and a 4% margin of error. The study is funded by Fondazione Pisa and approved by the Ethics Committee of Pisa (ID 26 533). All participants provide written informed consent prior to enrolment.

### PCCT scan

Technical details are summarized in *Table 1*. All examinations were performed on a dual-source photon-counting CT system (Naeotom Alpha; Siemens Healthineers®) with cadmium-telluride detectors and 0.25-s gantry rotation; collimation was 120 × 0.2 mm for the UHR coronary acquisitions and 144 × 0.4 mm for the high-pitch thoraco-cervical sweep and the 5-min delayed phase (QuantumPlus full-spectral mode).

**Table 1:** Acquisition protocol

Section	Details
Scanner	Dual-source photon-counting CT (Naeotom Alpha; Siemens Healthineers®) with cadmium-telluride detectors; 0.25-s rotation; collimation by phase: 120 × 0.2 mm for UHR coronary acquisitions and 144 × 0.4 mm for the high-pitch thoraco-cervical sweep and the 5-min delayed phase (QuantumPlus full-spectral mode).
Patient positioning	Supine with arms above the shoulders in a slightly oblique position to reduce overlap of carotids and mediastinum; baseline BP and HR measured; beta-blockers and/or nitroglycerin as indicated; single breath-hold with instruction not to swallow; beam-hardening mitigation with IMAR.
Acquisition phases	Non-contrast CAC: lung apices to L3, prospective ECG-triggered. UHR CCTA: 120 kVp, retrospective ECG-gated, pitch 0.2. High-pitch thoraco-cervical sweep (FLASH): ≤3.2 s, pitch 3.2, caudo-cranial (lung bases→orbital floor), prospective ECG-triggered. Late iodine enhancement (LIE, 5 min): heart + breasts, prospective ECG-triggered in diastole.
Additional acquisition	Low-dose expiratory scan to quantify air-trapping in COPD or in smokers with ≥20 pack-years.
Contrast protocol	Three-phase IV injection using iomeprol-400 or iopromide-370: Phase 1: 0.7 mL/kg undiluted contrast at 5–6 mL/s. Phase 2: 0.5 mL/kg contrast + 0.5 mL/kg saline at 3–4.5 mL/s. Phase 3: 50 mL saline. Total for a 70-kg patient: 84 mL (49 mL undiluted + 35 mL mixed phase).
Iodine dose	≈33.6 g (0.48 gI/kg) with iomeprol-400; ≈31.08 g (0.44 gI/kg) with iopromide-370.
Scan parameters	Tube potential: 120 kVp for CCTA; 140 kVp for medium-resolution thoraco-cervical acquisitions and the 5-min delayed phase. Collimation: 120 × 0.2 mm (UHR CCTA) and 144 × 0.4 mm (FLASH & LIE). CARE Dose 4D + CARE keV with reference IQ level 64; prospective ECG-triggering for CAC, FLASH, and LIE; retrospective ECG-gating for CCTA; combined DLP ≈ 740 mGy·cm (effective dose ≈ 11–13 mSv).
Image reconstruction	Coronaries: 0.2-mm slices at 0.1-mm intervals, 1024^2^ matrix, Bv60, QIR level 4, ZeeFree. Thoracic/cervical angiography: 0.4-mm slices at 0.2-mm spacing, 768^2^ matrix. Cine images for cardiac function: 1.5-mm slices, 1-mm interval across 20 cardiac phases (0–95%) on a 512 matrix.
Spectral post-processing	VMI at 40 keV (contrast amplification) and 70 keV (calcium subtraction); iodine maps for pulmonary perfusion and ECV; virtual non-contrast (VNC); iodine–VNC fusion images; pure lumen reconstructions for stenosis quantification.
Reconstruction software	ReconCT (syngo.via VB80C).

BP, blood pressure; CAC, coronary artery calcium; CCTA, coronary CT angiography; COPD, chronic obstructive pulmonary disease; ECG, electrocardiogram; ECV, extracellular volume; HR, heart rate; LIE, late iodine enhancement; DLP, dose-length product; UHR, ultra-high resolution; VMI, virtual monoenergetic images.

After venous access is established, patients are instructed about scanning steps and the expected sensations from contrast injection. Patients are positioned supine with arms above the shoulders. Arms are placed slightly oblique to reduce superimposition of carotids and upper mediastinum. The acquisition comprises ∼12 s for UHR coronary imaging, ∼5 s for automatic table repositioning, and a final ∼3-s high-pitch sweep encompassing lungs, breast parenchyma, and carotid arteries. The sequence is completed in a single breath-hold; patients are instructed to avoid swallowing. Baseline blood pressure and heart rate are measured before rate-control agents and/or nitroglycerin are administered,^11^ and patients are coached on breath-holding to optimize diagnostic quality.^11^

**Figure 2:**
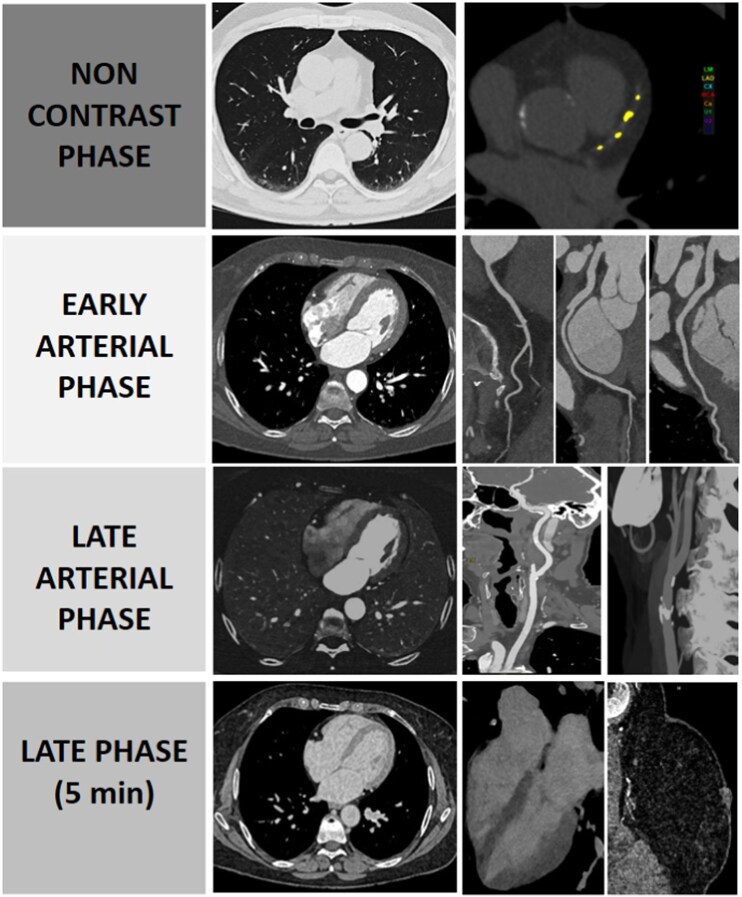
Scan phases. The protocol combines four sequential acquisitions: (i) non-contrast coronary artery calcium scoring, (ii) 0.2-mm ultra-high-resolution coronary CT angiography (70 kVp, retrospective ECG-gating), (iii) a < 3-s high-pitch helical sweep (pitch 3.4) from diaphragm to orbital floor, and (iv) 5-min delayed ECG-triggered late-iodine enhancement of heart and breasts.

The exam begins with a non-contrast spiral scan from lung apices to L3 to obtain coronary artery calcium (CAC) scores and a baseline assessment of pulmonary parenchyma. In smokers with ≥20 pack-years and/or chronic obstructive pulmonary disease (COPD), a low-dose expiratory acquisition is added to quantify air trapping. A delayed flash acquisition at 5 min after contrast is obtained in all patients with suspected/known structural heart disease and when early post-contrast images suggest breast lesions. An initial UHR coronary scan (cranio-caudal) is performed, followed by a rapid (≤3.2 s) FLASH-mode acquisition from lung bases to the orbital floor (caudo-cranial), during the same breath-hold. Five minutes later, a short Flash Scan late iodine enhancement (LIE) scan limited to heart and breasts with ECG triggering in diastole completes the protocol (*Figure 2*). Table time for all steps does not exceed 6–7 min.

The three-phase IV protocol aims to (i) achieve an early, high, uniform intraluminal peak for UHR-CCTA, (ii) maintain a flatter arterial plateau to cover the rapid high-pitch thoraco-cervical sweep without re-injection, and (iii) optimize contrast efficiency and reduce streak/beam-hardening by clearing residual contrast from the SVC and right heart, while providing predictable blood-pool kinetics for the brief delayed acquisition. Phase 1: 0.7 mL/kg undiluted iomeprol-400 or iopromide-370 at 5–6 mL/s. Phase 2: 1:1 contrast–saline mix (0.5 mL/kg contrast + 0.5 mL/kg saline) at 3–4.5 mL/s to prolong/flatten arterial enhancement across coronary, aortic, and supra-aortic territories. Phase 3: 50 mL saline flush to clear residual contrast and minimize artefacts. For a 70-kg patient, total contrast volume is 84 mL (49 mL undiluted + 35 mL from the mixed phase), yielding ∼33.6 g iodine (0.48 gI/kg) with iomeprol-400 and ∼31.08 g iodine (0.44 gI/kg) with iopromide-370.

Tube potential was 120 kVp for CCTA and 140 kVp for the medium-resolution thoraco-cervical acquisitions and the 5-min delayed phase. This higher kVp ensures robust thoraco-cervical penetration, mitigates beam-hardening/blooming in calcified vessels and stents, preserves Agatston scoring comparability, and stabilizes spectral outputs across body sizes. The CCTA is acquired at 70 kVp with retrospective ECG gating and dose modulation to exploit higher iodine attenuation for lumen/plaque assessment while limiting dose. For morphology/functional analysis, additional reconstructions use 1.5-mm slices at 1 × 0.7 mm intervals across 20 cardiac phases (0–95% in 5% steps) on a 512 matrix. Z-axis coverage uses 144 × 0.4 mm collimation; pitch is 0.2 during UHR coronary imaging and 3.2 during high-pitch (FLASH) acquisitions. Prospective ECG-triggered acquisition is applied for the non-contrast CAC scan, the immediate post-CCTA FLASH sweep, and the 5-min LIE scan. CARE Dose 4D and CARE keV are used with a reference image quality (IQ) level of 64. CARE Dose 4D adapts tube current in real time based on patient attenuation from the topogram, targeting the set IQ level (a vendor noise/SNR surrogate) rather than a fixed mAs; CARE keV is photon-counting–specific and optimizes dose while maintaining SNR/CNR at predefined keV settings. Exam-type presets (e.g. vascular) and manual kV options (70/90 kV for low-dose phases; 120/140 kV for high-resolution phases) are available; the ‘off’ mode is not used. These controls maintain image quality while adhering to ALARA principles. The combined dose-length product averages ∼740 mGy·cm, corresponding to an effective dose of ∼11–13 mSv, depending on individual characteristics.

Coronary data are reconstructed with 0.2-mm slices at 0.1-mm intervals on a 1024^2^ matrix (Bv60 kernel) with quantum iterative reconstruction (level 4). Each coronary dataset is additionally processed with ZeeFree to reduce stair-step artefacts and limit non-diagnostic segments. Thoracic and cervical angiographic data are reconstructed with 0.4-mm slices at 0.2-mm spacing on a 768^2^ matrix using lung, soft-tissue, or vessel-specific kernels as appropriate. Spectral CT enables virtual monoenergetic images (VMI) at 40 keV to amplify intravascular enhancement, 70 keV for calcium subtraction, and iodine maps for pulmonary perfusion and extracellular volume (ECV) quantification. Fusion of iodine maps with virtual non-contrast (VNC) images supports precise tissue iodine quantification. Pure lumen reconstructions facilitate stenosis quantification in heavily calcified plaques, particularly at the carotid bifurcation. All reconstructions are performed offline on ReconCT (syngo.via VB80C), which auto-selects the optimal cardiac phase and processes dual-energy batches.

To characterize image quality, signal-to-noise ratio (SNR), contrast-to-noise ratio (CNR), and sharpness were measured with standardized regions of interest (ROIs) and edge-profile analysis. On coronary images (0.2 × 0.1 mm, Bv60, 1024^2^) and mediastinal images (1.00 × 0.7 mm, Bv60, 512^2^), circular ROIs of ∼0.4 cm^2^ (≥100 pixels) were placed. For the heart, ROIs were drawn in epicardial fat (for fat HU) and the ascending aorta (for mean HU and SD); for the thorax, ROIs were placed in the descending aorta (mean/SD) and paraspinal muscle (mean). SNR was calculated as mean HU_Aorta/SD_Aorta, and CNR as (mean HU_Aorta—mean HU_Muscle)/SD_Aorta. Sharpness was derived from the full-width at half-maximum (FWHM) of the vessel edge spread: an attenuation line profile across the left main trunk was exported to ImageJ, fit with a Gaussian to obtain σ, and converted to FWHM using FWHM = 2.355*σ.

Automated algorithms quantify coronary plaque burden and diameter stenosis, while Agatston calcium scoring is derived from the initial non-contrast scan. Carotid stenoses are classified according to the Carotid Plaque-RADS system.^12^ Post-processing image reconstruction and quantitative analysis are performed using dedicated software (Syngo.Via, Siemens Healthineers, Erlangen, Germany). Plaque composition is analysed using spectral imaging data. Components are classified based on predefined Hounsfield unit (HU) thresholds: <60 HU for lipid-rich, 60–130 HU for fibrous, and >130 HU for calcified tissue. Lung nodules are categorized according to the Lung-RADS 2.2 system,^6^ emphysema is quantified below the −950 HU threshold, and iodine maps measure perfusion defects. Multi-phase reconstructions provide left ventricular end-diastolic and end-systolic volumes, ejection fraction and myocardial mass, and LIE datasets yield extracellular volume fractions after haematocrit correction.

## Results

### Main findings

Between January and June 2025, GPs invited 223 eligible individuals, and 172 were referred for PCCT. All 172 completed the examination without complications (i.e. no failed acquisitions or intolerance). Subjects undergoing PCCT had a median age of 62 years (interquartile range 54–68), and 56% were males. Thirty-four per cent had diabetes, 88% were current or former smoker, 66% had hypercholesterolaemia, 55% had hypertension, and 70% were overweight or obese. A history of atherosclerotic disease in the carotid arteries, the abdominal aorta or lower limb arteries was reported by 23%. Six per cent had known chronic obstructive pulmonary disease. One per cent had known lung nodules; additionally, 12 subjects (7%) had a history of malignant tumour (skin carcinoma, *n* = 3; breast cancer, *n* = 3; melanoma, *n* = 2; prostate cancer, *n* = 1; urothelial carcinoma, *n* = 1, thyroid cancer, *n* = 1; colon cancer, *n* = 1). Thirty-four per cent did not report any symptom, 44% complained on dyspnoea on effort, and just 14% had non-anginal chest pain. Median blood pressure levels were quite well controlled, and all subjects were in sinus rhythm. Median values of LDL-cholesterol, lipoprotein(a), and glycated haemoglobin were 103 mg/dL (78–127), 20 nmol/L (20–50), and 40 mmol/mol (36–47), respectively. Fifteen per cent of subjects were on aspirin or clopidogrel, and 43% on a lipid-lowering drug regimen.

Participants were classified according to the entry criteria into three groups: (i) individuals with diabetes for ≥ 10 years; (ii) non-diabetic current or former smokers; and (iii) non-diabetic, non-smoking individuals whose 10-year risk of death or myocardial infarction was ≥10%, as calculated by the Framingham Risk Score for hard CAD. The cohort included 59 diabetics (34%), 111 non-diabetics who were current or former smokers (65%), and only 2 (1%) with solely a risk score ≥10%. Among diabetics, 40 (68%) were current or former smokers (19 current and 21 former smokers). In the group of non-diabetics and current or former smokers, 81 (73%) were current and 30 (27%) were former smokers; furthermore, 39 (35%) had a 10-year risk ≥10% and 72 (65%) a risk <10%.

**Figure 3:**
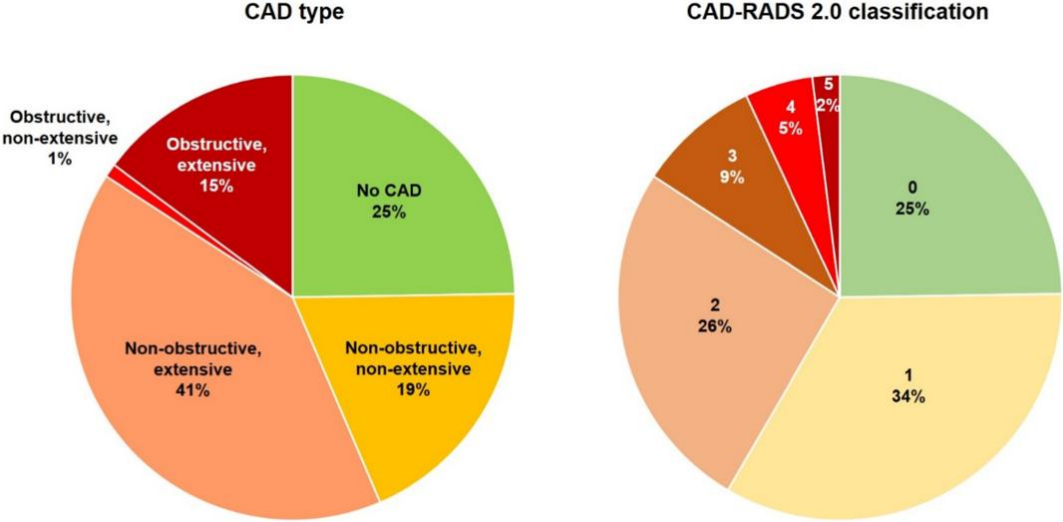
Classifications of coronary artery disease (CAD). CAD was classified according to Fuchs *et al.*^13^ and based on the CAD-RADS 2.0 classification.^14^

Any coronary atherosclerosis was found in 129 (75%). CAD could be classified as non-obstructive, non-extensive in 32 (19%), non-obstructive, extensive in 71 (41%), obstructive, non-extensive in 1 (1%), and obstructive, extensive in 25 (15%). The primary endpoint was thus met in 57% of subjects. CAD-RADS 2.0 score values were distributed as follows: 0 (no plaque or stenosis), *n* = 43 (25%); 1 (minimal stenosis or plaque with no stenosis), *n* = 58 (34%); 2 (mild stenosis, 25–49%), *n* = 45 (26%); 3 (moderate stenosis, 50–69%), *n* = 15 (9%); 4 (severe stenosis, 70–99% or left main stenosis ≥50% or 3-vessel disease with stenoses ≥70%), *n* = 8 (5%); 5 (total occlusion), *n* = 3 (2%) (*Figure 3*).

**Figure 4:**
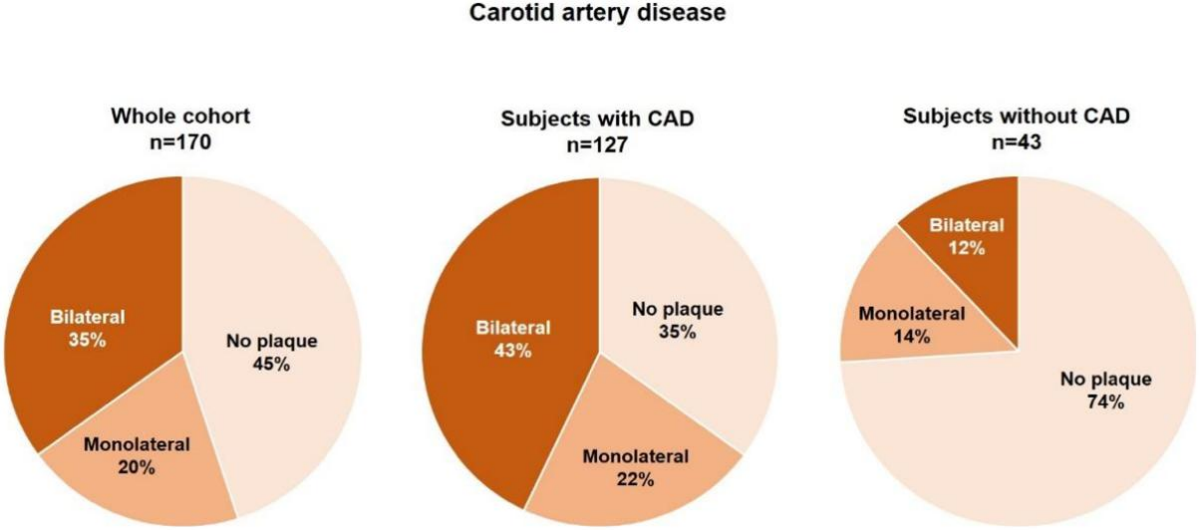
Prevalence of carotid artery disease. The percentages of subjects from the whole cohort (left) or without coronary artery disease (CAD; right) and no carotid plaques, monolateral or bilateral plaques are reported.

Carotid plaques could be assessed in all participants except two, whose scans were obscured by artefacts from dental implants. Carotid plaques were found in 94 subjects out of 170 (55%) and were bilateral in 60 (35%). One participant exhibited >70% stenosis of his left internal carotid artery. Another subject had a subocclusive plaque in the proximal segment of the left internal carotid artery. Among subjects without coronary atherosclerosis (*n* = 43), 11 (26%) displayed carotid artery disease, including 5 (12%) with bilateral disease (*Figure 4*). Forty-two subjects (24%) displayed plaques in the aorta or its branches, with ulcerated plaques in 3 (2%).

**Figure 5:**
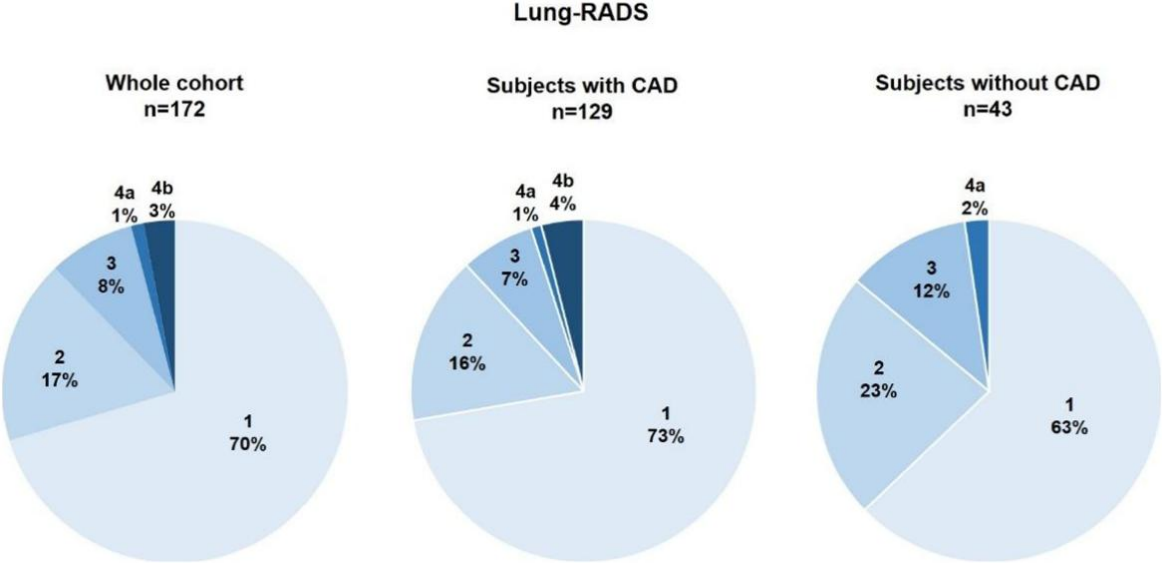
Lung-RADS scores. Results from the whole cohort (left) and the subgroup with coronary artery disease (CAD; centre) and without CAD (right) are reported.

Lung findings were classified as Lung-RADS 1 in 121 (70%), 2 in 30 (17%), 3 in 14 (8%), 4a in 2 (1%), and 4b in 5 (3%). When considering subjects with no evidence of coronary atherosclerosis, 27 (63%) scored 1, 10 (23%) scored 2, 5 (12%) scored 3, and 1 (2%) scored 4a (*Figure 5*). Forty-one individuals (24%) had parenchymal alterations suggestive of emphysema despite no history of COPD.

Incidental findings were frequent: thyroid nodules needing ultrasound (*n* = 34, 20%), breast lesions for specialist review (*n* = 13, 8%), lymphadenopathy >1 cm (*n* = 3, 2%), adrenal cortical hyperplasia/nodules (*n* = 4, 2%), and liver or kidney lesions warranting ultrasound (*n* = 12, 7%).

### Impact of PCCT scan on management

All participants with obstructive CAD were scheduled for exercise-stress myocardial perfusion scintigraphy, except for four individuals who were referred directly for invasive coronary angiography (ICA). In total, eight participants (5%) were referred to ICA. At the time of writing, seven subjects have undergone ICA, four have received percutaneous coronary intervention, and two coronary artery bypass graft surgery. The patient whose aortic valve area fell in the severe-stenosis range underwent an echocardiogram and surgical evaluation. Fifteen participants (9%) were referred for cardiac magnetic resonance imaging owing to unexplained hypertrophy, myocardial fibrosis, or fibro-fatty replacement. Two participants with suspected haemodynamically significant stenoses were referred for vascular surgery assessment. One patient with a subocclusive stenosis proceeded to carotid endarterectomy, whereas the other, whose stenosis was quantified as 65% on Doppler ultrasound, was scheduled for 6-month follow-up. All participants were also advised to undergo Doppler ultrasonography of the lower-limb arteries, to monitor their blood pressure regularly, and to engage in aerobic exercise in line with ESC guideline recommendations.^9^ A heart-healthy diet was recommended to everyone, with weight-loss counselling for those who were overweight or obese. Current smokers were strongly encouraged to quit. All participants with Lung-RADS category 3 to 4B nodules underwent multidisciplinary review by radiologists, pulmonologists, nuclear-medicine physicians, and thoracic surgeons. The remaining patients were managed according to Lung-RADS recommendations.^6^ Following ^18^F-FDG PET, one patient with two hypermetabolic nodules was referred for thoracic surgery. Forty-two subjects (24%) had radiological evidence of emphysema but no history of COPD and were referred to pulmonary function test and lung diffusion test, followed by a Pneumology visit. Twenty-five subjects have already performed these exams, and 8 (32%) have been diagnosed with COPD. Participants with incidental findings, such as thyroid nodules, abdominal lesions, or breast abnormalities, were directed to targeted investigations. Following thyroid ultrasound examination and needle aspiration biopsy, one patient was diagnosed with papillary thyroid cancer and underwent thyroidectomy.

Every participant with evidence of atherosclerosis in any vascular bed was prescribed an antiplatelet agent (aspirin unless contraindicated), if not already taking one. Lipid-lowering therapy was initiated or escalated in 67% of participants whose LDL-cholesterol levels exceeded ESC guideline targets^9^; each was asked to repeat a full lipid profile after two months to confirm target attainment. Patients with a history of poorly controlled hypertension, or with markedly elevated readings at the visit, were advised to start or intensify an antihypertensive regimen. All participants were referred to their general practitioners for blood-pressure assessment to ensure achievement of the <130/80 mmHg target.^9,15^ Four participants exhibited LV systolic dysfunction (LV ejection fraction 40–49%) with New York Heart Association class II symptoms. Two of them had N-terminal pro-B-type natriuretic peptide levels above the 125 ng/L diagnostic threshold for heart failure and were started on standard heart failure therapy^16^; the other two were scheduled for six-monthly cardiology follow-up.

### Six-month follow-up: first findings

In June and July 2025, the first 51 patients were contacted by telephone for the 6-month follow-up. Of the 42 patients reached, 35 (83%) reported adhering to all prescribed treatment changes, 4 did not adhere, and 2 reported partial adherence, primarily due to unwillingness to start aspirin and/or a statin. No adverse events attributable to the study procedures, treatment changes or further exams were reported. Thirty-five (83%) also reported undergoing at least some of the prescribed tests. The majority of patients (*n* = 39, 93%) reported measuring their blood pressure regularly. Seventeen (40%) initiated physical activity or increased its frequency. Among the 28 overweight or obese patients, 19 (68%) reported weight loss since the PCCT scan. Finally, of the 26 current smokers, 4 (15%) quit smoking and 11 (42%) reduced their daily number of cigarettes.

### PCCT scan: technical aspects

**Figure 6:**
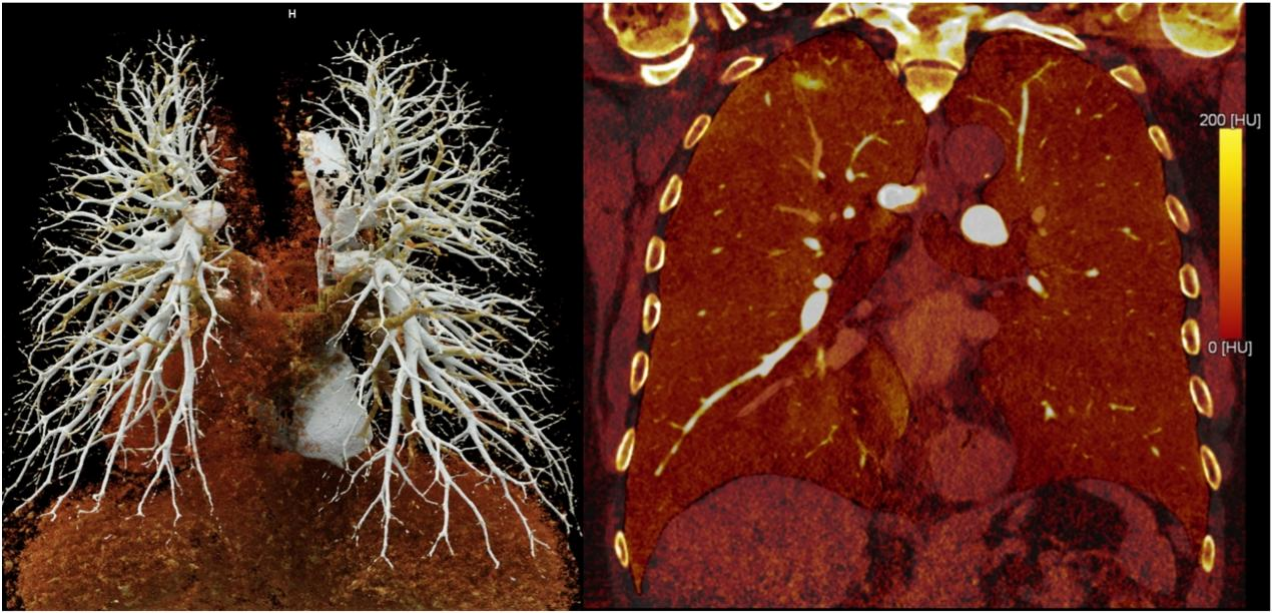
Volume rendering of the pulmonary vasculature from images at ultra-high resolution. Matrix 1024 × 2, slice thickness 0.2 × 0.1 mm. Iodine-based perfusion maps enable the detection of potential contrast-agent distribution defects within the lung parenchyma.

Of the 172 participants referred for PCCT, 38 (22%) received steroid premedication because of a history of drug allergy, and 69 (40%) received heart rate-lowering premedication. The exam was successfully completed in all subjects without any adverse event. The total effective dose was 12.6 mSv (10.5–17.1 mSv), of which 1.3 mSv (1.0–1.5 mSv) for the CAC score, 7.1 mSv (5.8–10.9 mSv) for the coronary CT angiogram, and 3.2 mSv (2.7–3.7 mSv) for the venous phase (for the lung and carotid scan). The late acquisition, performed in 55% of participants, added 0.6 mSv (0.0–0.9 mSv). *Figure 6* is an example figure demonstrating anatomical coverage and image quality.

In a prespecified subset of 40 consecutively enrolled participants (20 acquisitions performed at 70 kVp and 20 at 120 kVp), two independent readers performed ROI-based measurements of SNR and CNR in the UHR coronary and thoracic datasets. Image contrast in the coronary series was higher at lower tube potential: median (IQR) SNR and CNR were 9.71 [8.41–11.44] and 11.04 [9.30–12.71] at 70 kVp vs. 7.94 [6.77–8.99] and 9.32 [8.08–10.44] at 120 kVp. Thoracic SNR/CNR were broadly comparable across tube potentials (SNR 15.09 [12.72–18.60] vs. 14.70 [13.41–16.61]; CNR 13.66 [11.36–17.06] vs. 13.06 [11.99–14.67], 70 kVp vs. 120 kVp, respectively). Spatial resolution favoured the lower-kVp reconstructions, with narrower FWHM and smaller PSF values (FWHM 0.891 [0.828–0.981] mm at 70 kVp vs. 1.006 [0.925–1.188] mm at 120 kVp; PSF 0.378 [0.351–0.417] vs. 0.427 [0.393–0.505]). Inter-reader agreement was good for all metrics, with modest absolute differences for coronary measurements (CCT-HR SNR Δ 1.59 [0.64–2.35], CNR Δ 1.52 [0.66–2.70] at 70 kVp; SNR Δ 0.40 [0.21–0.82], CNR Δ 0.74 [0.34–1.14] at 120 kVp) and similar behaviour in thoracic datasets (SNR Δ 2.18 [1.10–3.20] and 0.70 [0.41–2.32]; CNR Δ 1.71 [0.89–2.69] and 0.80 [0.25–2.59], at 70 and 120 kVp, respectively).

## Discussion

We show that three examinations traditionally performed separately (non-contrast coronary calcium scoring, UHR-CCTA, and a rapid high-pitch thoraco-cervical sweep followed by a short LIE acquisition) can be consolidated into a single, multi-territory PCCT session without an apparent loss of diagnostic performance for the intended tasks. The combined dose-length product averaged ∼740 mGy·cm, corresponding to an effective dose of ∼12.6 mSv (10.5–17.1 mSv). A weight-adapted three-phase injection limited total iodine to ∼0.44–0.48 gI/kg. These values are comparable to a stand-alone coronary CTA and lower than the cumulative exposure/iodine load of performing three separate exams, framing the principal advantage as clinical consolidation rather than categorical dose reduction.

From a technical standpoint, the PCCT technology enable multi-energy output and UHR coronary imaging within a single breath-hold. Spectral reconstructions allow retrospective contrast-to-noise optimization for vascular, pulmonary, and soft-tissue tasks, while UHR coronary data improve depiction of calcified plaque and small-calibre segments. The same examination yields calcium scoring, plaque phenotype, Lung-RADS categorization, emphysema quantification, biventricular functional indices, and extracellular-volume mapping.

Beyond feasibility, we evaluated image quality in a prespecified subset (*n* = 40) using ROI-based SNR/CNR on the coronary UHR (120 × 0.2 mm) and thoracic series, and point-spread function/full width at half maximum (PSF/FWHM) for sharpness, with per-patient inter-reader variability. UHR CCTA at 120 kVp yielded high CNR and crisper edge definition, attributable to photon-counting detector granularity, ultra-high-resolution collimation, and low-keV spectral reconstructions, facilitating assessment of mixed/calcified plaque and distal segments where small gains in sharpness can influence visual stenosis grading. Thoracic image quality was maintained with the high-pitch sweep at 140 kVp, supporting our choice to prioritize penetration and spectral stability across the mediastinum, great vessels, and lung parenchyma without a material penalty in SNR/CNR. Inter-reader differences were modest, indicating good reproducibility and that spectral/UHR gains translate into consistent measurements. Overall, the image-quality profile supports the mixed-kVp strategy embedded in the protocol while completing the immediate phases within a single contrast bolus, with a brief additional breath-hold for the 5-min LIE acquisition.

The preliminary diagnostic yield supports clinical relevance. Any coronary atherosclerosis was present in 75% of participants; carotid plaques were seen in 55%, including bilateral disease in 35% of those with carotid atherosclerosis. Lung-RADS 3–4 categories accounted for ∼12%, and emphysema was detected in 24%. These findings translated into management changes: 5% underwent invasive coronary angiography, targeted vascular surgery referrals were issued for significant carotid disease, and pulmonary nodules were triaged per Lung-RADS. At 6-month follow-up, adherence to recommendations was high (83%), with reported lifestyle improvements and no adverse events attributable to study procedures or downstream testing.

Incidental extracardiac findings were common (thyroid nodules 20%, breast lesions 8%, lymphadenopathy 2%, adrenal 2%, indeterminate liver/kidney 7%) but were predominantly managed with non-invasive, guideline-directed steps under GP oversight. A pre-planned health-economic analysis will allow to judge net clinical value and potential over-medicalization.

Several limitations warrant mention. First, PCCT availability and capital costs may limit generalizability; the cost-effectiveness analysis will be important to inform adoption. Second, we report feasibility, yield, and early management impact, but resource use, patient-reported outcomes and clinical events will be evaluated after study completion and over a 5-year follow-up. Third, quantitative image-quality analysis is currently limited to a subset; expanded inter-observer and task-based metrics across the full cohort are being collected prospectively.

In summary, a single-session PCCT protocol can deliver comprehensive cardiothoracic risk information with consolidated radiation/iodine exposure, integrated reporting, and structured downstream pathways. The observed image-quality profile supports the technical premise of this consolidated workflow. The next step is to determine whether this strategy enables timelier prevention, is cost-effective, and ultimately reduces morbidity and mortality compared with usual care.

## Data Availability

Data are available upon reasonable request to the corresponding author.

## Lead author biography

Dr. Alberto Aimo, MD, PhD, FESC, FHFA, is a cardiologist at the Fondazione Toscana Gabriele Monasterio, a division of the Italian National Research Council, and a researcher at the Scuola Superiore Sant’Anna in Pisa, Italy. His research primarily focuses on heart failure and cardiomyopathies, with an emphasis on the use of biomarkers and advanced imaging for diagnosis and risk stratification, as well as drug and device therapies and structural interventions in heart failure.
